# The win ratio: a new approach to the analysis of composite endpoints in clinical trials based on clinical priorities

**DOI:** 10.1186/1745-6215-12-S1-A69

**Published:** 2011-12-13

**Authors:** Duolao Wang, Con Ariti, Tim Collier, Stuart Pocock

**Affiliations:** 1 Medical Statistics Department, London School of Hygiene and Tropical Medicine, London, UK

## Background

The conventional reporting of composite endpoints in clinical trials is unsatisfactory in that it emphasises each patient’s first event which is often of lesser clinical importance. The objectives of this talk are to introduce the concept of the win ratio for reporting composite endpoints to address this problem and to assess the new method by analysing real trials.

## Methods and results

Patients in the new and control treatment are formed into matched pairs based on their risk profiles. Consider a primary composite endpoint, e.g. cardiovascular (CV) death and heart failure hospitalisation (HF hosp) in heart failure trials. For each matched pair the new treatment patient is labelled a “winner” or a “loser” if it is known who had a CV death first. If that is not known, they are labelled a “winner” or “loser” if it is known who had a HF hosp first. Otherwise they are considered tied. The win ratio is the total number of winners divided by the total numbers of losers. If formation of matched pairs is impracticable then an alternative win ratio can be obtained by comparing all possible unmatched pairs. This method is insightfully illustrated by re-analyses of the EMPHASIS-HF, PARTNER B and CHARM trials.

## Conclusions

The win ratio is a powerful and easy to use method for reporting composite endpoints, and gives appropriate priority to the more clinically important event, e.g. mortality. We encourage its use in future trial design and analysis.

